# Tissue-Resolved Virome Analysis of Natural Chronic Bee Paralysis Virus Outbreak in *Apis mellifera* Reveals Segment Stoichiometry Shifts and Divergent Covert Infections

**DOI:** 10.3390/v18090938

**Published:** 2026-08-27

**Authors:** Nannan Li, Deya Wang, Huimin Yu, Zhihan Xu, Haoran Qin, Wei Li

**Affiliations:** 1 College of Life Sciences, Zaozhuang University, Zaozhuang 277160, China; linan_caas@163.com (N.L.); wangdeyasdny@163.com (D.W.); 2 College of Food Science and Pharmaceutical Engineering, Zaozhuang University, Zaozhuang 277160, China; 3 Zhucheng Animal Husbandry Development Center, Weifang 261061, China; yhmthrives@163.com; 4 College of Animal Science and Technology, Inner Mongolia Minzu University, Tongliao 028000, China; 18553697686@163.com; 5 College of Food and Drug Technology, Shandong Vocational Animal Science and Veterinary College, Weifang 261061, China

**Keywords:** chronic bee paralysis virus, *Apis mellifera*, natural outbreak, RNA1/RNA2 ratio, covert virome

## Abstract

Chronic bee paralysis virus (CBPV) is a bipartite positive-sense RNA virus that causes significant colony losses in honey bees, yet its tissue-level infection dynamics remain poorly characterized. We performed tissue-resolved virome analysis of two naturally infected *Apis mellifera* apiaries during a CBPV outbreak in Shandong, China. The apiaries shared a queen source and overwintering site but were separated by over 80 km at the time of sampling and developed contrasting disease patterns. Symptomatic foragers showing characteristic clinical signs of CBPV infection, including trembling, flightlessness, crawling, and hairless dark cuticle, and asymptomatic foragers were collected. Head, thorax, and midgut tissues were then dissected and used for RNA sequencing, yielding 36 RNA-seq libraries. In symptomatic bees, CBPV dominated the virome, with viral loads highest in heads and lowest in midguts. The RNA1/RNA2 copy ratio revealed a conserved tissue-specific gradient, shifting from RNA2 predominance in heads and thoraces to RNA1 enrichment in midguts (*p* = 0.024 for each apiary). This directional imbalance was validated by multiple controls and conserved across two genetically distinguishable CBPV populations, implicating the host tissue environment as the primary driver. In asymptomatic bees, the two apiaries harbored markedly different covert viromes, with CBPV dominant in the heads and thoraces of one apiary and near-exclusive Lake Sinai virus dominance in the other. These findings reveal that CBPV segment stoichiometry is modulated in a tissue-dependent manner in vivo and that viral genetic composition and covert virome composition can diverge notably between epidemiologically connected apiaries, providing additional insight into the infection biology of CBPV under natural conditions.

## 1. Introduction

Chronic bee paralysis virus (CBPV) is a globally distributed pathogen of the western honey bee (*Apis mellifera*) and a significant driver of adult worker mortality [1,2]. Clinically, infections present as two distinct syndromes: one characterized by trembling, flightlessness, and abdominal distension; the other by hairless, melanized bees that are actively rejected at the hive entrance [1,3,4]. While CBPV is frequently detected in apparently healthy colonies, viral loads are typically low in asymptomatic individuals but increase dramatically upon onset of clinical symptoms [5,6]. However, the composition of honey bee virus communities can vary over time and among geographic locations, and such variation has been reported across different seasons and regions [7,8,9,10]. The epidemiological triggers governing the shift from covert persistence to overt paralysis outbreaks remain elusive [11,12]. While environmental and host genetic factors are suspected to play a role, the viral determinants of this transition are comparatively underexplored, underscoring the need for high-resolution, tissue-resolved profiling to dissect the viral and host factors involved [13,14,15].

CBPV possesses a bipartite positive-sense RNA genome consisting of RNA1 and RNA2 [4]. RNA1 encodes the RNA-dependent RNA polymerase and associated non-structural proteins essential for replication, whereas RNA2 encodes the structural capsid proteins required for virion assembly [16]. This functional segregation raises the possibility that the two segments may not accumulate at equimolar ratios in vivo. Indeed, replication models derived from other bipartite RNA viruses suggest that segment accumulation can be independently regulated in a tissue- or host-specific manner [17,18].

PCR and quantitative RT-PCR (qPCR) are the mainstays of CBPV surveillance, but their inherent design imposes specific constraints [5,19,20]. These assays require prior sequence knowledge for primer design and are limited to predefined targets. Consequently, they lack the capacity to detect unsampled viral taxa beyond the selected targets [21]. Furthermore, their reliance on fixed primer sequences constrains robust detection of fast-evolving positive-sense RNA viruses, whose sequence landscapes shift across time and space [4]. Unbiased shotgun RNA sequencing provides a complementary approach by generating genome-wide data without a priori assumptions [22], thereby enabling the discovery of unanticipated viral diversity and accommodating sequence variation within a single analytical workflow.

We performed tissue-resolved RNA sequencing on symptomatic and asymptomatic workers from two apiaries that shared a genetic background and overwintering history yet developed contrasting disease outcomes. A conserved, tissue-dependent shift in CBPV segment stoichiometry was observed, with RNA2 predominating in heads and thoraces and the RNA1/RNA2 ratio rising significantly in the midgut. This pattern held across two genetically distinguishable CBPV populations, implicating the host tissue environment as the primary driver. In addition, asymptomatic bees from the two apiaries harbored markedly different covert viromes.

## 2. Materials and Methods

### 2.1. Study Design and Sample Collection

Two commercial *A. mellifera* apiaries, designated Apiary A and Apiary B, were sampled during a natural chronic bee paralysis virus (CBPV) outbreak in Weifang City, Shandong Province, China, in spring 2025. The two apiaries had overwintered within 1 km of each other. After spring relocation, Apiary A was moved approximately 80 km to a suburban apiary, while Apiary B remained at a stationary apiary. Both apiaries had experienced clinical signs consistent with chronic bee paralysis during the preceding year, including trembling, flightlessness, crawling, bloated abdomen, and hairless dark cuticle. No antibiotics or chemical treatments had been applied. Representative clinical signs of symptomatic foragers were recorded during sampling and are provided in Video S1.

From each apiary, one representative colony with the most typical symptoms was selected for sampling. For each colony, 15 symptomatic foragers were identified by characteristic CBPV signs, including trembling, flightlessness, crawling, and hairless dark cuticle, and 15 asymptomatic foragers were collected as returning bees that showed none of these clinical signs and exhibited normal flight and intact body hairs. Within each health status, the 15 bees were divided into three biological replicates of five bees each. Each biological replicate was dissected into head, thorax, and midgut tissues, and each tissue type was pooled separately for sequencing. This design yielded 18 libraries per colony and 36 libraries in total. Detailed information on sample grouping and processing is provided in Table S1.

Immediately after collection, whole foragers were flash-frozen in liquid nitrogen and transported to the laboratory for dissection into head, thorax, and midgut tissues. Dissected tissues were stored at −80 °C until RNA extraction.

### 2.2. RNA Extraction, Library Construction, and Sequencing

Total RNA was isolated using the RNApure Total RNA Kit (RN0302; Aidlab Biotechnologies Co., Ltd., Beijing, China). Ribosomal RNA was depleted, and sequencing libraries were constructed using the VAHTS mRNA-seq v2 Library Prep Kit for Illumina (NR601-01; Vazyme, Nanjing, China) according to the manufacturer’s protocol, with unique index sequences assigned to each sample. Paired-end sequencing (150 bp × 2) was performed on the Illumina NovaSeq platform, and a total of 3.35 billion clean reads were obtained after quality filtering.

### 2.3. Read Processing and Viral Sequence Identification

Raw reads were quality-filtered using fastp (version 1.0.1) [23] with default parameters to obtain clean reads. Clean reads were subjected to de novo assembly using Trinity (version 2.15.2) [24]. The assembled contigs were annotated against the NCBI non-redundant protein database using DIAMOND (version 2.1.9.163) [25] with an e-value threshold of 0.05. Contigs assigned to viral taxa were retained as candidate viral sequences.

To quantify virus-derived reads, clean reads from each library were mapped to the combined set of viral contigs using Bowtie2 (version 2.5.4) [26]. Clean reads were mapped to the *A. mellifera* genome (GCF_003254395.2_Amel_HAv3.1), and alignment counts were obtained with SAMtools (version 1.20) [27]. For targeted detection of 12 common honey bee viruses (Table S1), reads were aligned to viral references using Bowtie2 and counted with SAMtools.

For symptomatic bee libraries, consensus sequences of CBPV RNA1 and RNA2 were generated from the Bowtie2 alignments using SAMtools consensus with the following parameters: minimum depth 10, minimum mapping quality 20, minimum base quality 20, and heterozygous fraction 0.5. Full-length coverage was achieved for both CBPV segments in all symptomatic libraries, and consensus bases were supported by mapped reads at every position (Table S2). Libraries from asymptomatic bees were not subjected to consensus assembly due to their low CBPV read counts.

### 2.4. Quantification of CBPV Load and Segment Ratio

To quantify CBPV abundance relative to the host, the viral/host (V/H) read ratio was calculated by dividing the number of CBPV-mapped reads by the number of reads mapped to the *A. mellifera* reference genome for each library. The RNA1/RNA2 copy ratio was calculated as the number of reads mapped to RNA1 divided by the number of reads mapped to RNA2, corrected for the length difference between the two segments (RNA1: 3674 nt; RNA2: 2305 nt). To verify that the segment ratio was not biased by uneven coverage of untranslated regions, the ratio was recalculated using only reads mapping to the open reading frames of RNA1 and RNA2. Coverage uniformity across both segments was assessed by calculating per-base read depth and visualizing the distribution along the genome. Strand specificity was determined by separately counting reads mapping to the positive and negative strands of each segment using SAMtools.

### 2.5. Data Availability

Raw sequencing data have been deposited in the NCBI Sequence Read Archive under BioProject accession number PRJNA1496890. The assembled genome sequences of CBPV have been deposited in GenBank under accession numbers PZ718828-PZ718863.

## 3. Results

### 3.1. Study Design and CBPV Dominance in Symptomatic Bees

**Figure 1:**
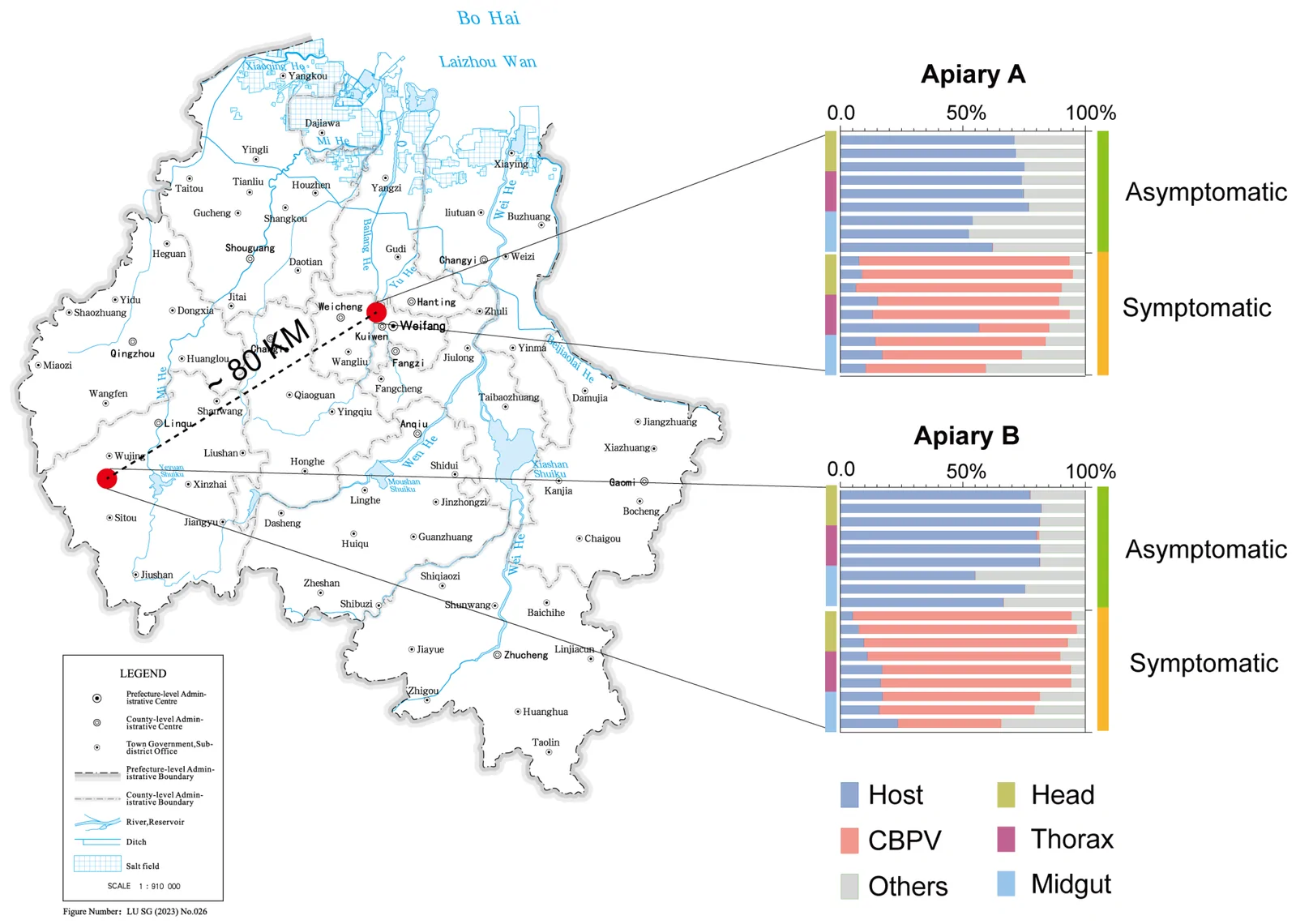
Study design and sampling overview. (**Left**): geographic locations of the two study apiaries in Weifang, Shandong (~80 km apart). (**Right**): CBPV abundance across all 36 libraries. CBPV abundance across all libraries (**right panel**) was quantified by mapping clean reads to the CBPV reference genomes MZ821953.1 (RNA1) and MZ821954.1 (RNA2) and the honey bee reference genome GCF_003254395.2, respectively, with proportions calculated as the percentage of total clean reads. Samples were grouped by tissue (head, thorax, midgut), health status (symptomatic, asymptomatic), and apiary (Apiary A, Apiary B). Proportions were calculated as the percentage of total clean reads assigned to CBPV or host, with the remaining reads classified as Others. Each bar represents one biological replicate consisting of five pooled bees. Head, thorax, and midgut were obtained from the same five bees per replicate. *n* = 3 biological replicates per group.

In spring 2025, a natural CBPV outbreak was identified in two commercial *A. mellifera* apiaries in Weifang, Shandong (Figure 1, left). Despite sharing queens from the same breeding stock and an overwintering site (<1 km), after spring relocation, they were spatially isolated (~80 km apart) and developed different epidemiological patterns: Apiary A showed a focal outbreak with a large number of infected beehives, while Apiary B displayed diffuse, low-intensity clinical signs with consistently lower individual losses.

To investigate the virome correlates of these contrasting outcomes, we collected symptomatic and asymptomatic foragers from each apiary and performed tissue-resolved metatranscriptomic sequencing of the head, thorax, and midgut (36 libraries total; average 93.17 million clean reads per library; Table S1). Targeted screening against 12 known bee viruses, combined with de novo assembly and nr database annotation (no novel insect viruses were identified), revealed that CBPV dominated the symptomatic virome in both apiaries, accounting for an average of 94.57% of total viral reads and 70.98% of all reads (Figure 1, right). Pairwise comparisons of CBPV loads across all four apiary/symptom combinations (Apiary A/B × symptomatic/asymptomatic) confirmed that symptomatic bees harbored significantly higher CBPV levels than asymptomatic bees in both apiaries (Mann–Whitney U test, *p* < 0.0001 for both), with no significant difference between symptomatic bees of the two apiaries (Figure S1).

### 3.2. Genetic Relatedness of CBPV Strains Between Apiaries

To determine whether the two epidemiological patterns were associated with distinct viral strains, we generated consensus sequences for both CBPV genomic segments from all 18 symptomatic libraries. Within Apiary B, sequences were highly homogeneous, with pairwise identities exceeding 99.6% for both RNA1 and RNA2. Within Apiary A, RNA1 was similarly conserved (>99.0%), while RNA2 showed slightly broader variation (96.51–99.97%).

**Figure 2:**
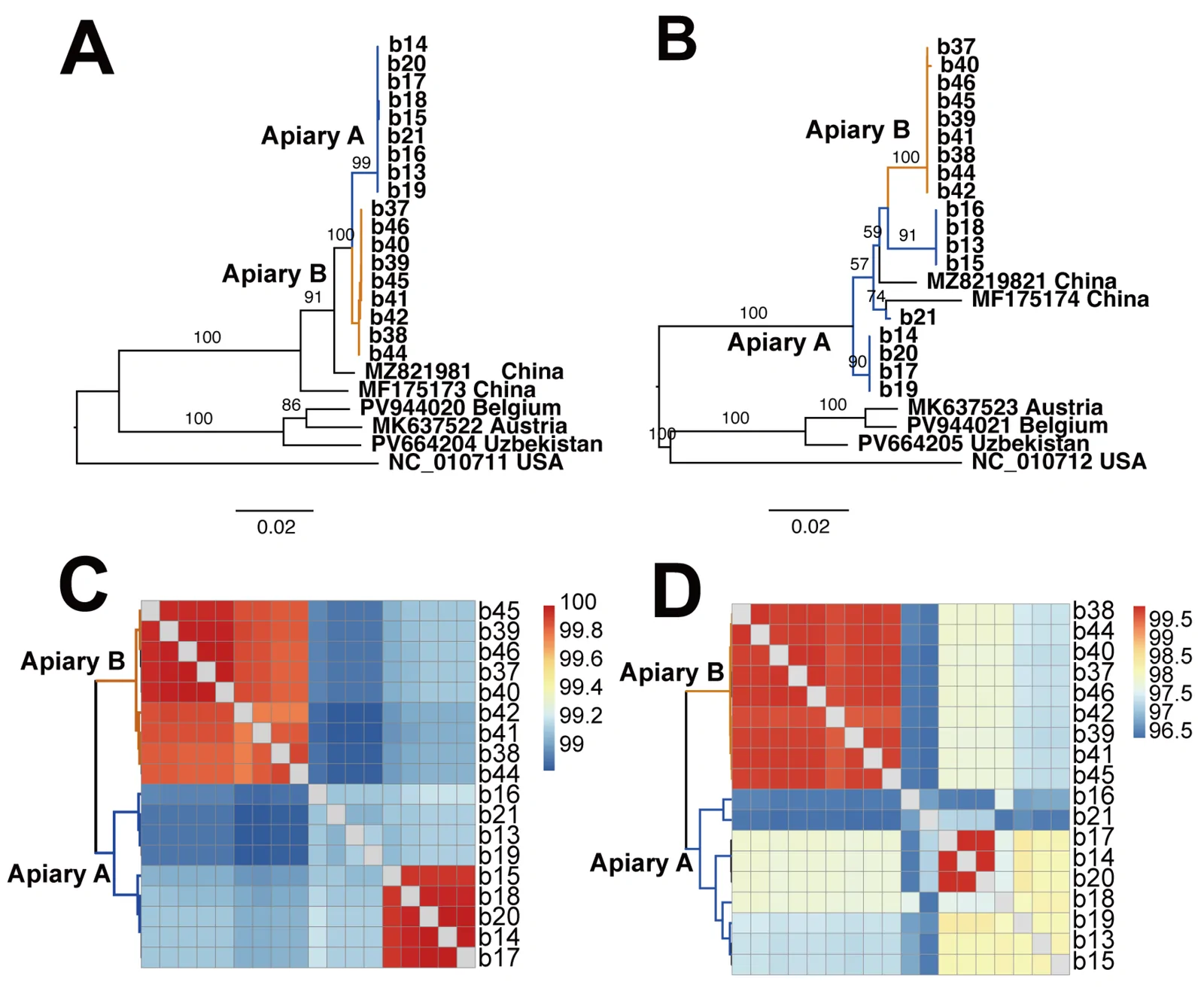
Phylogenetic and clustering analysis of CBPV RNA1 and RNA2 consensus sequences from 18 symptomatic libraries. (**A**,**B**) Maximum-likelihood phylogenetic trees of CBPV RNA1 (**A**) and RNA2 (**B**), including the closest GenBank sequences and representative isolates from other countries. One representative consensus sequence from each apiary, derived from libraries b21 and b44, was compared against the NCBI nucleotide database by BLASTN. For RNA1, the closest match was MZ821981.1 from Heilongjiang, China, with nucleotide identities of 97.85% and 98.71% for the two representatives. For RNA2, the closest match was MF175174.1 from Hebei, China, with nucleotide identities of 96.28% and 98.25%, respectively. Published CBPV sequences from GenBank are labeled with their accession numbers and countries of origin. Bootstrap support values are indicated at the corresponding nodes. (**C**,**D**) Clustering trees based on pairwise identity values among the 18 consensus sequences from this study, shown for RNA1 (**C**) and RNA2 (**D**).

Inter-apiary pairwise identities ranged from 98.84% to 99.13% for RNA1 and from 96.43% to 98.01% for RNA2 (Supplementary Tables S2 and S3). To place these sequences in a broader phylogenetic context, we included CBPV reference sequences from GenBank, comprising those with the highest nucleotide identity to our consensus sequences (MZ821981.1 for RNA1 and MF175174.1 for RNA2) as well as representative isolates from other countries. Maximum-likelihood phylogenies of RNA1 (Figure 2A) and RNA2 (Figure 2B) showed that our sequences grouped with CBPV sequences from China and were clearly separated from representative isolates from other countries. In the RNA1 phylogeny, sequences from Apiary A and Apiary B each formed distinct clusters, while in the RNA2 phylogeny, the overall resolution was lower, and some Apiary A sequences did not form a single well-supported group, with moderate-to-low bootstrap support at several internal nodes. In both phylogenies, our sequences grouped with CBPV sequences from China and formed a distinct monophyletic clade that was clearly separated from representative isolates collected from other countries. Clustering trees based on pairwise identity values (Figure 2C for RNA1; Figure 2D for RNA2) recapitulated the same topological patterns (Tables S3 and S4). Collectively, these data indicate that the two apiaries harbored closely related but distinguishable CBPV populations, with Apiary B being genetically homogeneous and Apiary A exhibiting greater intra-apiary diversity, particularly in RNA2, which encodes the capsid protein.

### 3.3. CBPV Load and Segment Stoichiometry in Diseased Bees

**Figure 3:**
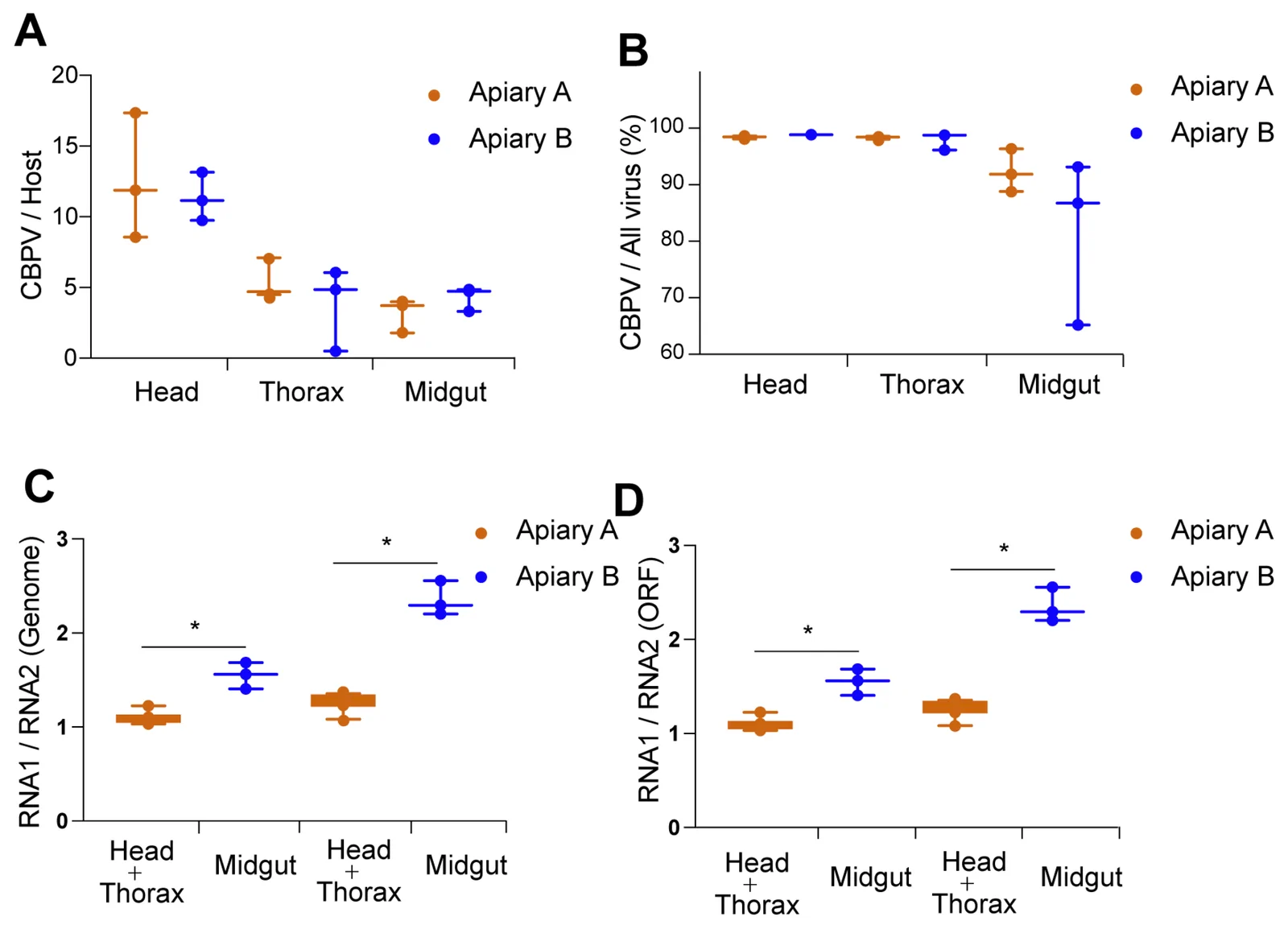
CBPV load and segment stoichiometry in symptomatic bees. (**A**) CBPV abundance normalized to host reads (V/H ratio) across head, thorax, and midgut tissues of symptomatic bees from Apiary A and Apiary B. Each dot represents one biological replicate (*n* = 3 per tissue per apiary). (**B**) CBPV proportion within the viral community, shown as the percentage of total viral reads (reads mapped to all identified viral genomes) in each library. (**C**) RNA1/RNA2 read ratios calculated using reads mapping to the full-length reference genomes. (**D**) RNA1/RNA2 read ratios calculated using reads mapping exclusively to the coding regions (ORFs). For both (**C**,**D**), ratios were calculated by first dividing the read counts mapped to each segment by the corresponding segment length and then dividing the length-normalized RNA1 value by the length-normalized RNA2 value. Statistical significance was assessed by the Mann–Whitney U test. * *p* < 0.05; exact *p* = 0.024 for the comparison between midgut and head/thorax within each apiary. Each dot represents one biological replicate prepared from five pooled bees. *n* = 3 per tissue per apiary.

We profiled all 18 diseased bee pools, with 9 pools from each of the two sampled colonies, across head, thorax, and midgut. When normalized to host reads (V/H ratio), CBPV abundance displayed tissue-dependent variation: heads consistently showed the highest values (Apiary A median 11.88, range 8.57–17.34; Apiary B median 11.15, range 9.74–13.16), thoraces intermediate (Apiary A median 4.69, range 4.49–7.11; Apiary B median 4.85, range 0.51–6.06), and midguts the lowest (Apiary A median 3.71, range 1.79–3.99; Apiary B median 4.73, range 3.32–4.85) (Figure 3A). One Apiary B thoracic sample (b42) showed a markedly low V/H ratio of 0.51. Its paired head sample (b39) exhibited a V/H ratio comparable to those of other diseased bees, ruling out inadvertent inclusion of an asymptomatic individual. Whether this outlier reflects genuine biological variation or a technical artifact could not be determined; we therefore retained it in all analyses. In parallel, CBPV dominated the viral community, accounting for ≥96.12% of total viral reads in all diseased heads and thoraces, while in midguts its dominance remained evident but less absolute (65.20–96.30%) (Figure 3B). Collectively, these two measures confirm that CBPV was the overwhelmingly dominant virus in symptomatic bees across both apiaries and tissues.

Given the tissue-dependent variation in CBPV abundance, we next assessed whether the relative proportions of RNA1 and RNA2 were similarly tissue-specific by calculating a length-corrected RNA1/RNA2 copy ratio. Pairwise comparisons among head, thorax, and midgut detected no significant differences (Figure S2), likely owing to the limited number of replicates per group (*n* = 3). Given the similar RNA1/RNA2 ratios in head and thorax within each apiary, together with their shared neural composition and status as primary CBPV infection sites, we pooled head and thorax data for subsequent comparison with midgut. In heads and thoraces of both apiaries, the ratio was consistently below 1.0 (Apiary A head and thorax median 0.68, range 0.65–0.77; Apiary B head and thorax median 0.80, range 0.68–0.85), revealing a subtle but consistent stoichiometric bias toward RNA2. In the midgut, however, the ratio shifted significantly toward RNA1 (Figure 3C). This tissue-specific elevation was statistically significant in both apiaries (Mann–Whitney U test, Apiary A *p* = 0.024, Apiary B *p* = 0.024), with Apiary A midguts approaching equimolarity (median 0.98, range 0.88–1.06) and Apiary B midguts reaching a clear RNA1 excess (median 1.44, range 1.38–1.61). Thus, despite apiary-level differences in absolute values, the directional shift towards a higher RNA1/RNA2 ratio in the midgut relative to heads and thoraces was conserved.

To confirm that this tissue-specific imbalance was not driven by uneven UTR coverage, we recalculated the ratio using reads mapping exclusively to the ORFs. The ORF-based ratios faithfully recapitulated the tissue-specific pattern, including the slight RNA2 excess in heads and thoraces (Figure 3D), confirming that the imbalance originates from the protein-coding regions. To further exclude the possibility of tissue-specific internal degradation, we examined the coverage depth distribution across both segments. Coverage was uniform along RNA1 and RNA2 in all tissues, with no evidence of internal dropouts in midgut samples (Figure S3A–D). Additionally, strand-specific analysis revealed that positive-sense genomic RNA constituted >98% of all viral reads in every tissue, and the minimal fraction of negative-strand intermediates (<2%) did not differ between midguts and other tissues (Figure S3E). Taken together, these validation analyses confirm that the elevated RNA1/RNA2 ratio in the midgut is a robust biological feature of CBPV infection, potentially reflecting tissue-dependent differences in replication dynamics or segment-specific packaging efficiency. The median RNA1/RNA2 ratio in Apiary B midguts (1.44) was higher than that in Apiary A (0.98), although the small sample size (*n* = 3 per group) precluded formal statistical comparison.

### 3.4. CBPV Covert Infection Profiles Differ Between Apiaries

**Figure 4:**
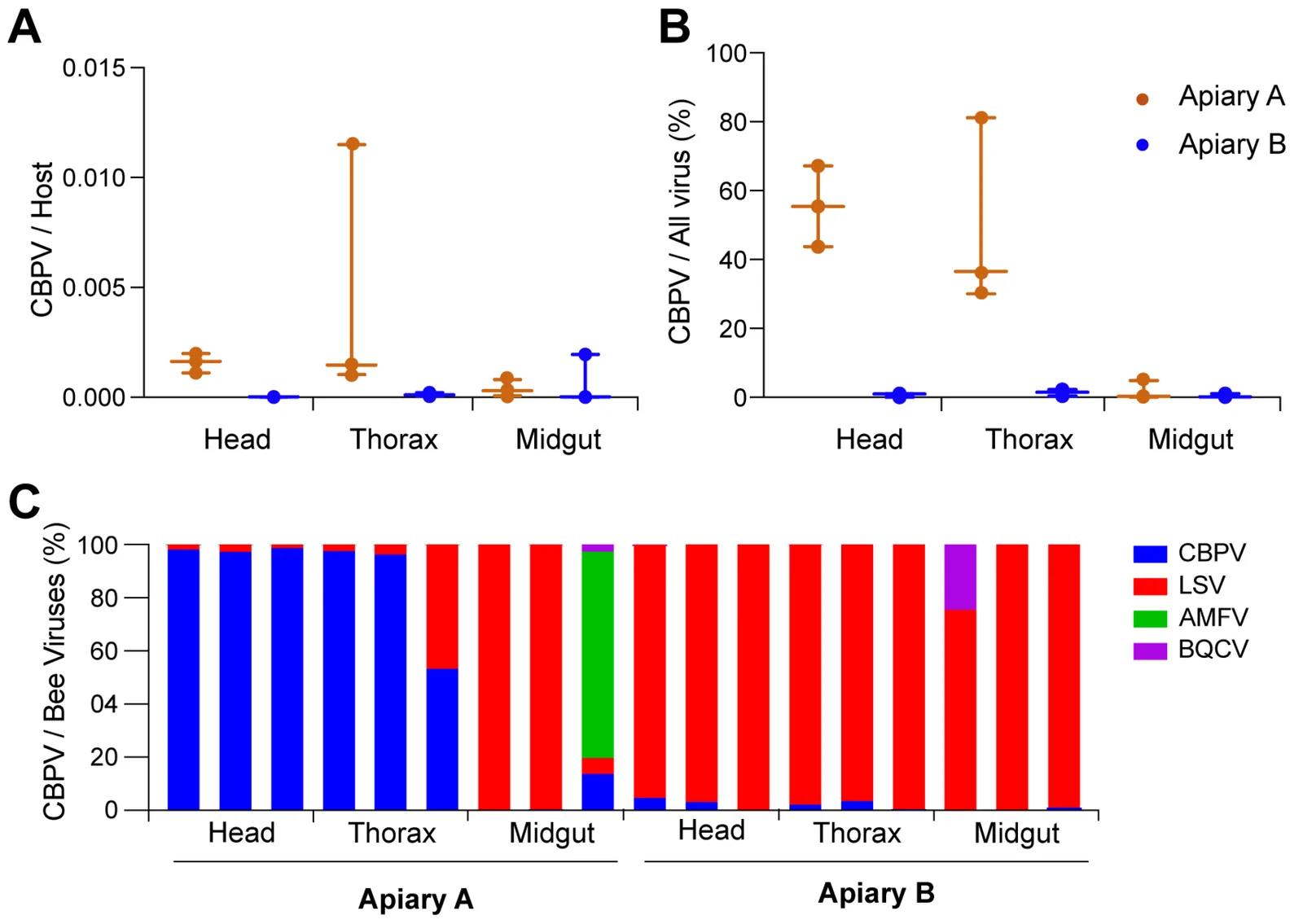
CBPV covert infection profiles differ between apiaries. (**A**) CBPV abundance normalized to host reads (V/H ratio) in asymptomatic bees from Apiary A and Apiary B across head, thorax, and midgut tissues. (**B**) CBPV proportion within the viral community, shown as the percentage of total viral reads in each asymptomatic library. Each dot represents one biological replicate prepared from five pooled bees. *n* = 3 per tissue per apiary. (**C**) Viral community composition in asymptomatic libraries from both apiaries. The stacked bars show the relative abundance of the four dominant viruses: CBPV, LSV, AMFV, and BQCV. The remaining eight targeted bee viruses were detected at negligible levels and are therefore not visualized; complete read counts for all 12 targeted viruses are provided in Table S1. Each bar represents one biological replicate consisting of five pooled bees. Head, thorax, and midgut were obtained from the same five bees per replicate. *n* = 3 biological replicates per group.

In contrast to the extensive viral dominance observed in symptomatic bees (Figure 1), all asymptomatic bees maintained CBPV at low levels, with V/H ratios remaining at least two orders of magnitude below those of symptomatic bees. The two apiaries displayed markedly distinct covert infection profiles, with Apiary A exhibiting consistently higher CBPV prevalence than Apiary B across both the V/H ratio (Figure 4A) and CBPV proportion within the virome (Figure 4B). Relative to the host transcriptome, Apiary A heads showed V/H ratios approximately 100-fold higher than Apiary B heads (median 1.63 × 10^−3^, range 1.11 × 10^−3^–2.00 × 10^−3^ vs. median 1.43 × 10^−5^, range 8.40 × 10^−6^–2.53 × 10^−5^). Within the viral community, CBPV accounted for 43.75–67.16% of total viral reads in Apiary A heads and 30.02–81.14% in thoraces, whereas in Apiary B it constituted 0.06–1.01% in heads and 0.37–2.26% in thoraces (Figure 3B). These differences were most pronounced in the head and thorax. In asymptomatic midguts, CBPV loads remained substantially lower than in the head and thorax across both apiaries (Apiary A V/H: 7.01 × 10^−5^–8.12 × 10^−4^; Apiary B V/H: 4.43 × 10^−6^–1.95 × 10^−3^, with b9 showing an elevated value of 1.95 × 10^−3^ relative to the other asymptomatic midguts despite its asymptomatic status). Its viral community dominance was also consistently below 5% (0.004–4.89% across both apiaries).

In all asymptomatic samples from Apiary B, LSV dominated the virome, with LSV2 as the predominant genotype (Table S5) and one library showing BQCV at approximately 20% of viral reads. In Apiary A, CBPV dominated the viral community in heads and thoraces, while in midguts LSV predominated in two samples and AMFV in the third. Thus, the two apiaries harbored distinct covert viromes, with CBPV dominance in Apiary A heads and thoraces contrasting with near-exclusive LSV dominance across all tissues in Apiary B (Figure 4C).

## 4. Discussion

In spring 2025, two apiaries that shared a queen source and overwintering site developed divergent CBPV outbreak patterns. This natural contrast provided an opportunity to examine virome features associated with disease severity. Two findings emerged from the tissue-resolved comparison.

First, a tissue-specific imbalance in the copy ratio of the two CBPV genomic segments was conserved across both apiaries, with the RNA1/RNA2 ratio consistently below 1 in the head and thorax but significantly elevated in the midgut. This ratio shift was validated by ORF-only mapping, uniform coverage, and strand-specific analysis, confirming that it is not an artifact. Because the directional shift was conserved across two genetically distinct CBPV populations while the consensus sequences within each apiary were highly homogeneous across tissues, the host cellular environment, rather than viral genotype, most likely drives the imbalance.

This tissue-dependent segment imbalance is reminiscent of the segment-specific regulation observed in the Nodaviridae, where RNA1 replicates autonomously while RNA2 requires a specific 3′ stem-loop for replicase recognition [4,28]. Here, even though consensus sequences were nearly identical across tissues, the RNA1/RNA2 ratio still shifted significantly between the head/thorax and midgut. We note that our consensus sequences lack independent 3′ end verification; we cannot formally exclude the possibility that mutations in the terminal regions alter RNA secondary structure, which in turn influences segment accumulation in a tissue-dependent manner. Nonetheless, the conservation of this directional shift across two genetically distinguishable CBPV populations, combined with the high homogeneity of consensus sequences within each apiary, suggests that host tissue microenvironments can modulate segment abundance in a manner largely independent of consensus-level genetic variation.

The biological significance of this stoichiometric plasticity remains to be determined. The midgut is a critical site for CBPV entry, replication, and fecal–oral transmission [14,28]. It is plausible that an RNA1 excess in the midgut supports replication in this immunologically active environment, while the slight RNA2 excess in the head and thorax may reflect a higher demand for structural proteins at sites of virion assembly and shedding, such as the hypopharyngeal and salivary glands [5,28]. However, alternative mechanisms, including differential RNA stability, packaging efficiency, or translational regulation, are equally possible. In other segmented and multipartite viruses, segment copy number can directly modulate gene expression. In the multipartite Faba bean necrotic stunt virus, each segment’s copy number correlates with its cognate mRNA concentration in a host-dependent manner [17,18]. Whether CBPV similarly tunes its RNA1/RNA2 ratio to adjust the balance of replicase and structural proteins in a tissue-specific manner remains an open question, but the robustness of the pattern across two independent apiaries warrants further investigation.

In addition to this tissue-specific segment stoichiometry, the covert virome of asymptomatic workers differed markedly between the two apiaries, with CBPV dominant in Apiary A heads and thoraces and LSV nearly exclusive in Apiary B. This divergence cannot be readily explained by our data. A larger CBPV reservoir in Apiary A’s asymptomatic workers may have facilitated viral amplification when conditions favoring overt infection arose. Conversely, one possible interpretation is that high LSV abundance may be associated with reduced CBPV replication. However, this remains a speculative hypothesis, and the present data do not allow it to be tested directly. LSV is highly prevalent in honey bee populations worldwide and has been associated with colony strength, yet its biology remains poorly understood [29,30,31,32]. LSV and CBPV share distant RdRp similarity [29], and whether this distant relatedness has any functional relevance is unknown. Moreover, honey bee virus communities are known to vary over time and among geographic locations, and the present comparison is based on a single sampling time point and a limited number of biological replicates. Therefore, the observed virome differences may reflect temporal or geographic variability rather than a stable biological interaction between LSV and CBPV. Disentangling host from viral drivers is complicated by the confounding of viral genotype, colony identity, and apiary environment [11,15]. Controlled co-infection experiments will be required to determine whether LSV can actively exclude CBPV and whether covert virome composition causally influences disease outcome. Broader spatiotemporal sampling and controlled co-infection experiments will be required to determine whether LSV and CBPV interact in vivo and whether such interactions affect disease progression.

Several limitations should be noted. First, only two apiaries from a single region and time point were examined, and broader spatiotemporal sampling is needed to assess the generality of these findings. Second, although three replicates per group were sufficient to detect the conserved stoichiometric pattern, they provided limited statistical power for inter-apiary comparisons of absolute ratio values.

## 5. Conclusions

In summary, this study reveals that CBPV segment stoichiometry varies across tissues in a conserved pattern, implicating the host tissue environment as the primary driver. It also demonstrates that both viral genetic composition and covert virome composition can diverge detectably between epidemiologically connected apiaries. These findings identify segment ratio as a tissue-associated variable in CBPV infection and suggest that covert virome structure may be associated with disease outcome, although broader spatiotemporal sampling is needed to assess the generality of these observations.

## Data Availability

Raw sequencing data have been deposited in the NCBI Sequence Read Archive under BioProject accession number PRJNA1496890. The assembled genome sequences of CBPV have been deposited in GenBank under accession numbers PZ718828-PZ718863.

## Supplementary Materials

The following supporting information can be downloaded at https://www.mdpi.com/article/10.3390/v18090938/s1. Figure S1: Pairwise comparisons of CBPV loads across four apiary/symptom combinations; Figure S2: RNA1/RNA2 ratios in head, thorax, and midgut of bees from Apiary A and Apiary B; Figure S3: Technical validation of RNA1/RNA2 ratio measurements in symptomatic bees; Table S1: Sample metadata, sequencing statistics, and viral read counts; Table S2: Coverage and mean sequencing depth of CBPV RNA1 and RNA2 in the 18 symptomatic libraries; Table S3: Pairwise nucleotide identity matrix of CBPV RNA1 consensus sequences from 18 symptomatic libraries; Table S4: Pairwise nucleotide identity matrix of CBPV RNA2 consensus sequences from 18 symptomatic libraries; Table S5: Read counts of eight Lake Sinai virus (LSV) genotypes in each library; Video S1: Clinical signs of symptomatic foragers used in this study.

## Abbreviations

The following abbreviations are used in this manuscript:

AMFV	*Apis mellifera* filamentous virus
BQCV	Black queen cell virus
CBPV	Chronic bee paralysis virus
LSV	Lake Sinai virus
ORF	Open reading frame
RdRp	RNA-dependent RNA polymerase
RNA-seq	RNA sequencing
V/H ratio	viral/host read ratio
