# Supplementary material for: Tissue-Resolved Virome Analysis of Natural Chronic Bee Paralysis Virus Outbreak in Apis mellifera Reveals Segment Stoichiometry Shifts and Divergent Covert Infections
Source: Viruses. 2026 Aug 27;18(9):938. doi: 10.3390/v18090938 (PMC13612030; doi:10.3390/v18090938)
Supplement: Supplementary file 1 [file viruses-18-00938-s001.zip › Supplementary Figures.pdf]

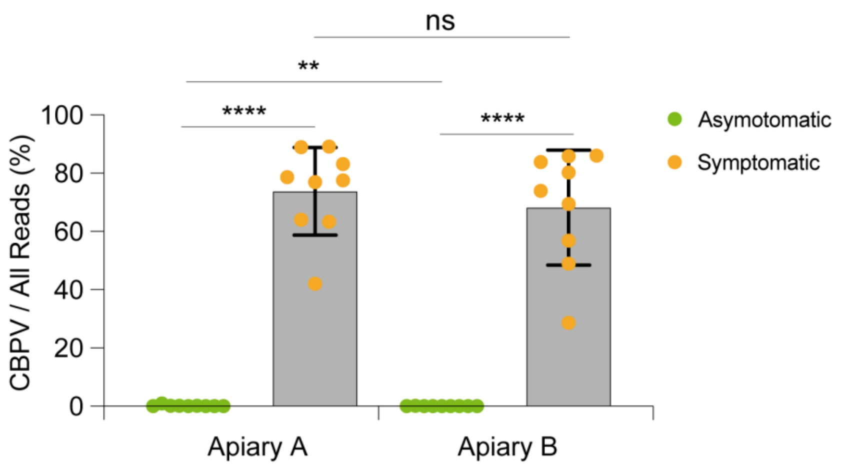

Figure S1. Pairwise comparisons of CBPV loads across four apiary/symptom combinations. Bars represent medians with interquartile ranges; individual data points are overlaid. Statistical significance was assessed by Mann–Whitney U test. **p < 0.01, ****p < 0.0001; ns, not significant.

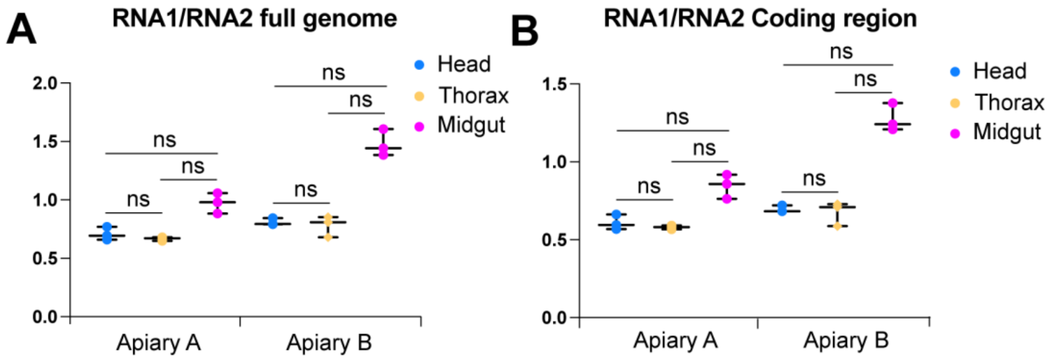

Figure S2. RNA1/RNA2 ratios in head, thorax, and midgut of bees from Apiary A and Apiary B. (A) Full-genome RNA1/RNA2 ratios. (B) Coding-region RNA1/RNA2 ratios. Data are presented as mean ± SEM, with individual data points overlaid. Statistical significance was assessed by Kruskal–Wallis test followed by Dunn's multiple comparison test. ns, not significant (p ≥ 0.05).

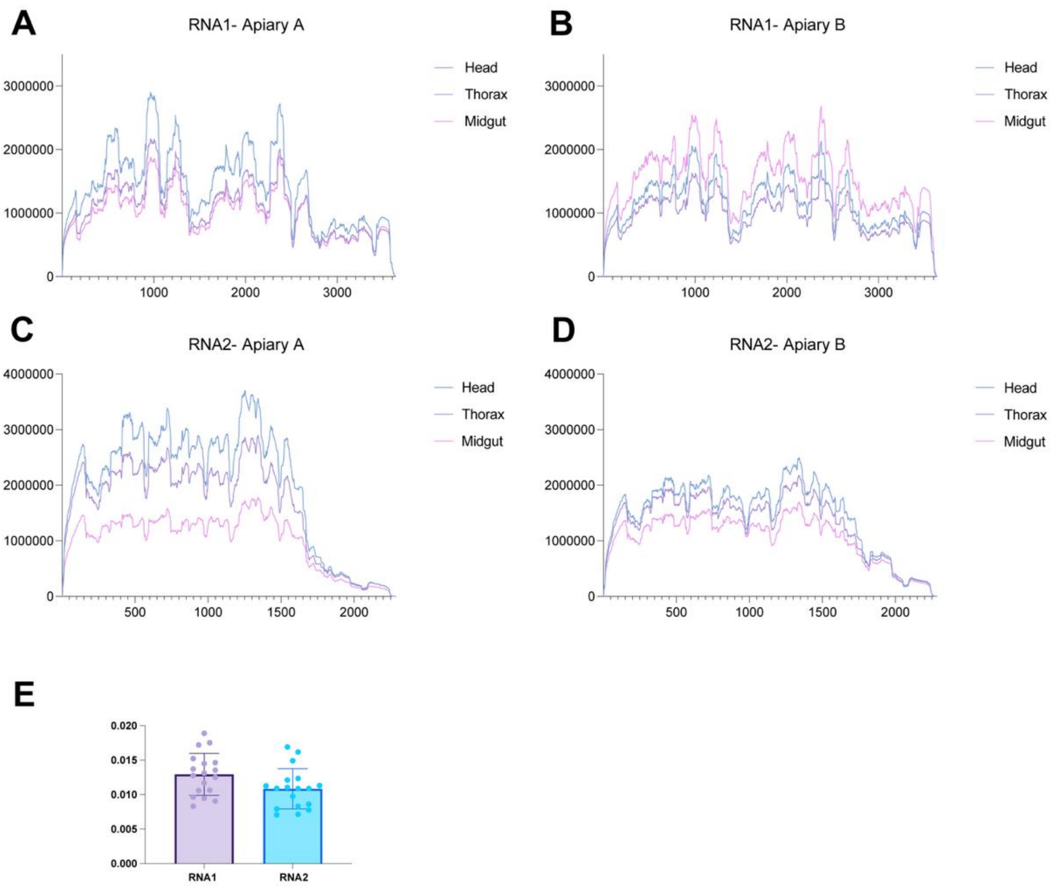

Figure S3. Technical validation of RNA1/RNA2 ratio measurements in symptomatic bees. (A–D) Coverage depth plots for CBPV RNA1 and RNA2 across head, thorax, and midgut tissues from Apiary A and Apiary B. Panels (A) and (B) show coverage along RNA1 for Apiary A and Apiary B, respectively; panels (C) and (D) show coverage along RNA2 for Apiary A and Apiary B, respectively. Coverage depth was calculated using Samtools. (E) Ratio of negative-sense to positive-sense CBPV RNA across all tissues and both apiaries. The reads mapped to negative-sense to positive-sense chains were determined by filtering reads based on SAM flag values using Samtools.
